# Plasma–Induced Modification Mechanisms of PET Films: Correlated Evolution of Topographical Features and Surface Chemical States

**DOI:** 10.3390/nano16100615

**Published:** 2026-05-17

**Authors:** Yang Wang, Ying Yang, Jinlian Hu, Yuanyuan Lu, Xiaoyu Hao, Jun Zheng

**Affiliations:** Key Laboratory of Green Fabrication and Surface Technology of Advanced Metal Materials, Ministry of Education, Anhui University of Technology, Ma’anshan 243002, China

**Keywords:** RF plasma treatment, polyethylene terephthalate (PET), surface functionalization, topographical modification, wettability regulation

## Abstract

The effects of RF plasma treatments using different gases (Ar, O_2_, and N_2_) and processing parameters on the surface wettability of polyethylene terephthalate (PET) films were systematically investigated. Atomic force microscopy (AFM) and X–ray photoelectron spectroscopy (XPS) were employed to characterize the evolution of surface topography and chemical composition. While all treatments enhanced hydrophilicity, the magnitude of improvement and the governing mechanisms were gas-dependent. Among them, O_2_ plasma treatment exhibited the most pronounced effect: under optimal conditions (20 W, 80 s), the water contact angle (WCA) was reduced to 3.7°, indicating a superhydrophilic surface. This enhancement was primarily attributed to a substantial increase in surface oxygen content (O/C ratio) and the incorporation of strongly polar oxygen-containing functional groups, such as C=O and COOH. N_2_ plasma offered moderate improvement via nitrogen-containing groups, while non-reactive Ar plasma relied primarily on physical etching, yielding the smallest enhancement. Analysis revealed that wettability evolution was dominated by increased polar surface energy from chemical functionalization, with surface roughness playing a synergistic role. These results demonstrate that optimizing plasma gas and parameters effectively controls PET wettability through the coupled regulation of surface chemistry and topography.

## 1. Introduction

Metallized film is a functional material fabricated by depositing a thin metal layer onto a polymer substrate through vacuum evaporation or magnetron sputtering [1]. It is widely used in fields such as food packaging, film capacitors, solar cells, and lithium batteries [2,3,4]. This material combines the flexibility, lightweight nature, and processability of the polymer substrate with the conductivity, electromagnetic interference shielding capability, and decorative properties provided by the metallic layer. However, due to the low surface energy of polymers, the deposited metal clusters typically follow the Volmer–Weber growth mode, resulting in a porous microstructure of the metal layer and poor adhesion to the substrate [5,6]. To enhance the surface energy of polymers, plasma treatment is widely employed as a surface modification technique prior to metal deposition [3,7]. Effective modification is achieved by altering the microstructure, surface roughness, and chemical composition of the nanoscale surface layer of the polymer without compromising its bulk properties. The underlying mechanism can be described as follows: the polymer surface is bombarded by energetic ions accelerated within the electric field, leading to scission of molecular chains and the generation of a substantial number of radicals. These radicals can subsequently react with adjacent radicals on the same chain to form various chemical bonds, or they can bond with radicals on neighboring chains, resulting in the formation of cross-linked structures [8,9,10]. Additionally, they are capable of reacting with active species present in the plasma to achieve surface functionalization [11]. Currently, plasma treatment has been extensively investigated as an effective method for polymer surface modification. It can significantly enhance the surface energy of polymer substrates, improve the density of deposited metal layers, and strengthen film adhesion. Pelagade et al. [12] reported that the PET surface energy was increased from 48.6 mJ/m^2^ to 83.2 mJ/m^2^ by Ar plasma treatment as the exposure time was extended from 5 min to 50 min, primarily attributed to the introduction of polar functional groups (e.g., C-O, C=O). Similarly, Kurdi et al. [13] found that chemical bonds such as Al-N-C and Al-N-C-O formed at the interface when Al was deposited on NH_3_ plasma-pretreated PP, which remarkably enhanced the film adhesion. In another study, Kouicem et al. [14] demonstrated that Ar + O_2_ plasma pretreatment of PMMA substantially improved the adhesion of sputtered Al films. This improvement was attributed to the combined effects of chemical bonding (via introduction of C=O and C-O groups) and mechanical interlocking, resulting from the increased surface roughness. In our previous work, the PP substrate was pretreated by air plasma, followed by the deposition of Au film via magnetron sputtering. It was found that the plasma pretreatment significantly increased the nucleation density of Au clusters and suppressed the Volmer–Weber growth mode [15].

Based on the chemical activity, gases used for plasma treatment can be classified into two categories: reactive gases (e.g., O_2_, N_2_, NH_3_) and non-reactive gases (e.g., Ar, He). The former primarily enhance the polymer hydrophilicity through surface functionalization by introducing polar groups, whereas the latter improve wettability mainly via physical etching, which alters surface roughness [16,17]. However, regarding the comparative effectiveness of plasma treatments using different gases, divergent results were found in the existing literature. For instance, in the study by Mirabedini et al. [18], PP films were treated with Ar and O_2_ plasmas at 10 W, 30 W, and 50 W. The optimal modification was achieved at 50 W, where PP films pretreated with Ar plasma exhibited a smaller contact angle and superior wettability compared to those treated by O_2_ plasma. Moreover, in a study by Shi et al. [19], N_2_ and Ar plasmas were employed to modify an acrylic-based Polymer Multilayer (PML), revealing that N_2_ treatment significantly outperformed Ar in improving wettability. In direct contrast to the aforementioned findings on Ar and O_2_, Junkar et al. [20] demonstrated that for PET substrates, O_2_ plasma yields vastly superior wettability compared to Ar plasma. These conflicting results may originate from variations in processing equipment, as well as the inherent chemical composition and dissociation pathways of the specific polymers involved. Such discrepancies are particularly pronounced for complex polymers comprising three or more elements. Taking PET as an example, the benzene ring and ester group in its structure lead to a particularly complex reaction mechanism with the plasma species [21]. Owing to its excellent mechanical and thermal properties, PET has found widespread practical applications in flexible electronics [22], packaging and printing [23], and biomedical devices [24]. To further meet the demanding interfacial adhesion requirements of these applications, extensive research in recent years has been devoted to specific plasma treatments on PET. For instance, Pelagade et al. [12] treated PET films with Ar plasma and observed a gradual decrease in the water contact angle (WCA) with prolonged treatment time. After 50 min of Ar treatment, the WCA dropped to 15°. Similarly, the aforementioned study by Junkar et al. [20] found that O_2_ treatment rapidly reduced the PET contact angle to 19°, while N_2_ treatment lowered it to 24°. In contrast, Vesel et al. [25] reported a contact angle of only 30° following O_2_ treatment. Consequently, a simultaneous comparative analysis under identical experimental conditions is still lacking.

While independent treatments and specific gas-pair comparisons have demonstrated the effectiveness of plasma modification, previous studies frequently relied on single-liquid contact angle evaluations and lacked a unified comparative framework. Because surface wettability is synergistically governed by both chemical composition and physical roughness, a comprehensive investigation utilizing dual-liquid contact angle measurements under identical processing conditions is highly necessary to explicitly correlate these surface variations. Furthermore, existing studies struggle to quantitatively separate the contributions of physical etching and chemical functionalization. Research has long attributed the decreased water contact angle to a synergistic effect of newly generated polar groups and increased roughness, often qualitatively relying on the classical Wenzel equation [26]. However, because water is a highly polar liquid, its contact angle is extremely sensitive to both surface chemistry and topography. Analyzing water contact angles alone results in highly coupled effects, making it mathematically impossible to isolate the exact contribution of surface roughness.

To address this gap, this study systematically evaluates the effects of Ar, O_2_, and N_2_ plasma treatments on PET substrates. By conducting all modifications within the same RF plasma system across systematically varied power and time parameters, equipment-induced systemic variations are minimized. Atomic Force Microscopy (AFM) and X-ray Photoelectron Spectroscopy (XPS) are employed to quantify the morphological evolution and the relative concentration of surface polar functional groups, respectively. Subsequently, the surface free energy is decoupled into its dispersion component ($\gamma_{s}^{d}$) and polar component ($\gamma_{s}^{p}$) utilizing diiodomethane as a non-polar reference liquid. We then correlate the decoupled $\gamma_{s}^{d}$ with the AFM-derived surface area difference (SAD), and the $\gamma_{s}^{p}$ with the XPS-identified chemical states. This approach clarifies the distinct modification mechanisms of different plasma atmospheres, establishing a reliable correlation among micro-level topography, surface chemistry, and macroscopic wettability.

## 2. Materials and Methods

### 2.1. Sample Preparation

**Figure 1:**
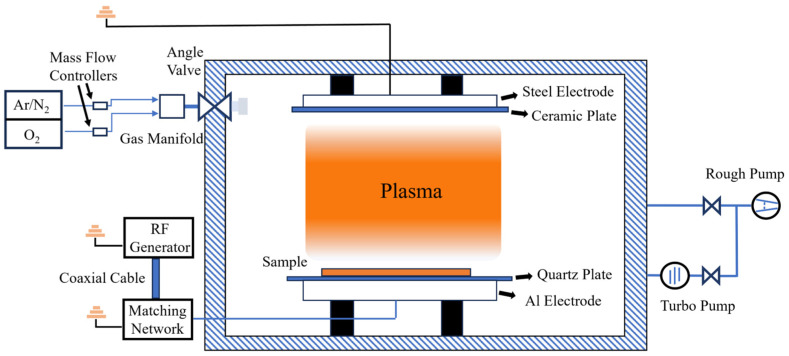
Schematic diagram of plasma treatment device.

In the present work, biaxially oriented PET films with a thickness of 37 μm were selected as substrates (purchased from Suzhou Dongxuan Plastic Products Co., Ltd., Suzhou, China). Pretreatment of the PET films was conducted by a self-made radio-frequency (RF) plasma device; the schematic diagram is shown in Figure 1. The main components of this device, including the vacuum system and gas control system, were based on a high-vacuum multifunctional thin-film deposition system (JSZY350, Beijing Technol Science Co., Ltd., Beijing, China). The vacuum system consisted of a turbo pump and a rough pump, achieving a base pressure of 5.0 × 10^−5^ Pa. Plasma was generated by a dielectric barrier discharge (DBD) system, which consisted of an RF power supply (RSG500, Ruisejel Electronics Technology Co., Ltd., Changzhou, China)and parallel-plate electrode configurations. The RF power supply operated at a frequency of 13.56 MHz with an output accuracy of ±2 W. An RF matching network was integrated into the system to ensure that the reflected power remained below 2 W throughout the experiment. RF power was applied to the lower aluminum electrode, while the upper steel electrode was grounded. The parallel-plate configuration featured an effective area of 100 cm^2^ with an inter-electrode gap of 69 mm. In order to avoid the electrodes being sputtered by the plasma, the two plates were covered with a ceramic glass plate and a quartz plate, respectively. The gas delivery system comprised two independent supply lines, each precisely regulated by a dedicated mass flow controller (MFC). The gas lines were connected to a common gas manifold and the gas was introduced into the vacuum chamber via a vacuum angle valve. For more detailed information on the apparatus, please refer to the literature [27].

Prior to the experiment, the PET samples were cut into 70 × 70 mm squares and affixed to a quartz plate using 3M high-temperature tape. The chamber was then evacuated to a base pressure of ≤2.0 × 10^−3^ Pa, followed by the introduction of high-purity (99.999%) treatment gas (Ar, O_2_, or N_2_) until a stable working pressure of 1 Pa was established. Plasma treatment was subsequently carried out at fixed RF power and duration settings, with the detailed parameters summarized in Table 1, Table 2 and Table 3.

### 2.2. Sample Characterization

A self-made contact angle goniometer was employed to test the contact angles of PET films before and after plasma treatment. To strictly avoid the influence of the aging effect and ensure experimental consistency, all contact angle measurements were performed immediately after the plasma treatments. Measurements were performed with 8 μL droplets of deionized water (self-prepared in the laboratory, polar) and diiodomethane (99%, containing copper stabilizer; McLean Biochemical Technology Co., Ltd., Shanghai, China, nonpolar) as the test liquids. To minimize measurement variability, contact angles were measured at five randomly selected locations on each sample, and the final result was derived from the arithmetic mean value. The surface energy ($\gamma_{s}$) and its dispersive ($\gamma_{s}^{d}$) and polar ($\gamma_{s}^{p}$) components were calculated using Young’s equation and Owens–Wendt–Rabel–Kaelble (OWRK) method, as shown in Equations (1) and (2), respectively.

(1) $$\gamma_{s}={\;\gamma }_{l}\cos\theta +\gamma_{\mathrm{sl}}$$

(2) $$\gamma_{\mathrm{sl}}=\gamma_{l}+{\;\gamma }_{s}-\;2\left(\sqrt{\gamma_{s}^{d}\cdot {\;\gamma }_{l}^{d}}+\sqrt{\gamma_{s}^{p}\cdot {\;\gamma }_{l}^{p}}\right)$$

Here, $\gamma_{s}$ and $\gamma_{l}$ denote the surface energy of solid and liquid, respectively. $\gamma_{\mathrm{sl}}$ is the solid–liquid interfacial energy. θ presents the contact angle. The symbols $\gamma_{s}^{d}$ and $\gamma_{l}^{d}$ stands for the dispersion components of the solid and liquid surface energy, respectively; $\gamma_{s}^{p}$ and $\gamma_{l}^{p}$ correspond to their respective polar components. For deionized water, $\gamma_{l}^{d}$ is 27.19 mN·m^−1^ and $\gamma_{l}^{p}$ is 45.61 mN·m^−1^ at 20 °C; for diiodomethane, the corresponding values are $\gamma_{l}^{d}$ = 50.8 mN·m^−1^ and $\gamma_{l}^{p}$ = 0 mN·m^−1^ at 20 °C [28,29].

The surface topography and roughness were analyzed by atomic force microscopy (AFM, Bruker Icon, Billerica, MA, USA) operating in tapping mode with a tip radius of 8 nm. Meanwhile, the chemical states were examined using X-ray photoelectron spectroscopy (XPS, Thermo Scientific Nexsa, Waltham, MA, USA) with monochromatic Al Kα radiation (12 kV, 1486.6 eV), and the spectra were deconvoluted using Avantage software (Version 6.9.0) to identify functional groups and quantify their relative contents.

## 3. Results

### 3.1. Surface Properties of PET Films After Ar Plasma Treatment

#### 3.1.1. Effect of Ar Plasma Treatment on Surface Energy of PET Films

**Figure 2:**
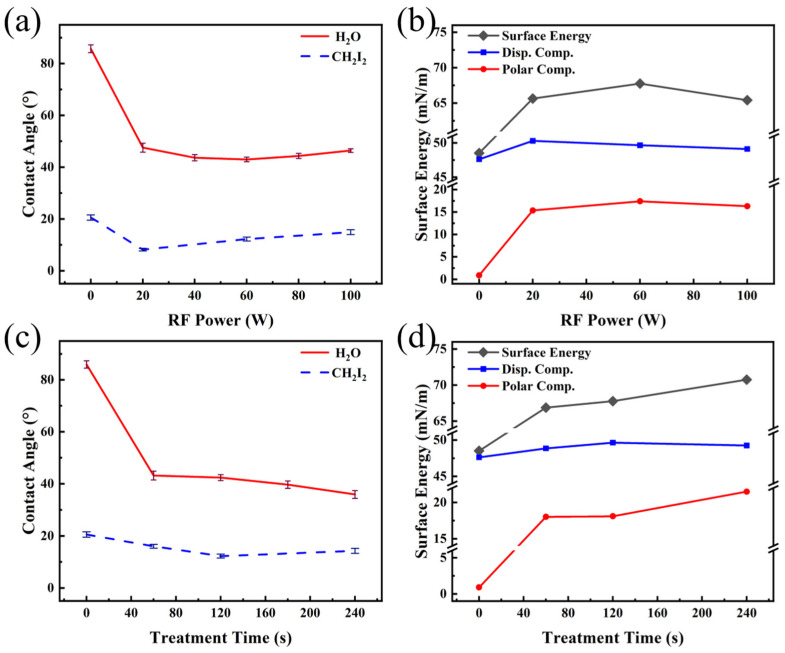
Contact angle and surface energy of PET films as a function of Ar plasma treatment parameters: (**a**,**b**) RF power and (**c**,**d**) treatment time.

The effect of Ar plasma treatment on the wetting properties of PET films is shown in Figure 2. The corresponding water droplet photographs are provided in Supplementary Figures S1–S4. As illustrated in Figure 2a, the water contact angle (WCA) of the untreated film was 85.9°, which was in good agreement with the result reported in ref. [21]. After plasma treatment, the WCA decreased significantly as the RF power was increased from 0 W to 20 W, whereas further increases in RF power resulted in minimal change. The diiodomethane contact angles (DCA) were measured at RF powers of 20 W, 60 W and 100 W. A non-monotonic dependence was observed: the angle decreased to a minimum at 20 W and subsequently increased with further elevation of the RF power. Based on the OWRK method and Young’s equation, the surface energy of the PET films, as well as their dispersive and polar components, was calculated. As shown in Figure 2b, the polar component increased sharply with RF power up to 20 W, beyond which it remained nearly constant. This trend correlated well with the variation in WCA described above, confirming that the rise in the polar component was the main reason for the enhanced surface wettability with water. Meanwhile, the dispersive component reached its maximum at 20 W and exhibited a slight decrease at higher RF powers. This behavior was consistent with the trend observed for the DCA, indicating that the decrease in the DCA could be primarily attributed to the increase in the dispersive component of the PET surface.

To investigate the effect of treatment time on the surface wettability of PET, a systematic study was conducted at a fixed power of 60 W. The results are presented in Figure 2c. As shown, the WCA decreased continuously with prolonged treatment time, reaching a minimum of 35.9° at 240 s. Similarly, the DCA exhibited a declining trend with increasing treatment duration, attaining its lowest value at 240 s. The surface energy of the PET films, along with their dispersive and polar components under different treatment times were calculated, as shown in Figure 2d. The influence of treatment time exhibited a trend similar to that of RF power in Figure 2a,b: the variation in the polar component aligned with the evolution of the WCA, while the change in the dispersive component corresponded to the trend of the DCA. Under the combined effect of both components, the surface energy of PET reached a maximum value of 70.75 mN·m^−1^ at a treatment time of 240 s.

#### 3.1.2. Effect of Ar Plasma Treatment on the Topography of PET Films

**Figure 3:**
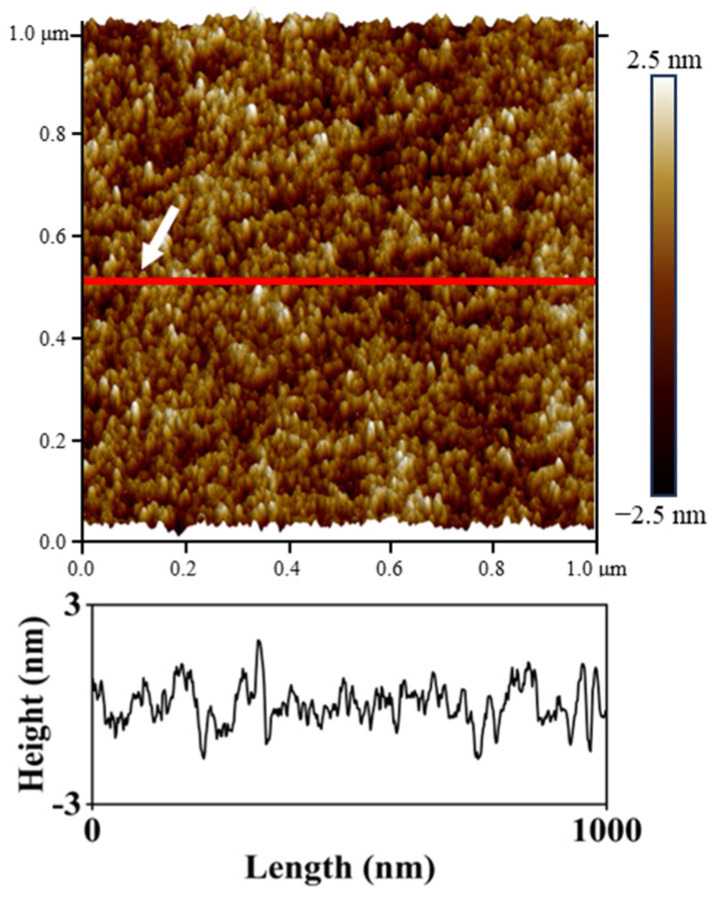
Surface topography of the untreated PET film. The white arrow and red line indicate the location of the height profile used for surface analysis. The same convention is applied to all subsequent AFM images.

Figure 3 illustrates the topography of the untreated PET film with a scanning area of 1 μm × 1 μm. The surface was characterized by a high density of nanoscale peaks. Cross-sectional profile analysis further revealed that these features possessed a low overall height, resulting in a relatively smooth surface. The arithmetic average roughness (Ra) was measured as 0.58 nm.

**Figure 4:**
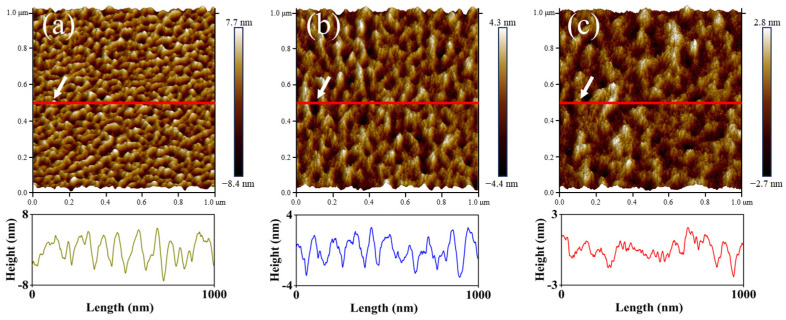
Topography of PET films treated with Ar plasma at different power levels: (**a**) 20 W; (**b**) 60 W; (**c**) 100 W.

Figure 4 presents the AFM topography of PET samples treated with Ar plasma at 20 W, 60 W, and 100 W. It can be seen that the PET surface exhibited continuous ridge-like features when treated at 20 W. As the power was increased to 60 W, both the density and height of these ridges decreased. When the power was further raised to 100 W, the ridge-like morphology gradually disappeared, resulting in a comparatively flat surface. The surface roughness, derived from the AFM data, exhibited a consistent decrease with increasing RF power: it dropped from 1.99 nm at 20 W to 1.01 nm at 60 W, and further declined to 0.63 nm at 100 W.

#### 3.1.3. Effect of Ar Plasma Treatment on the Surface Chemical State of PET Films

**Figure 5:**
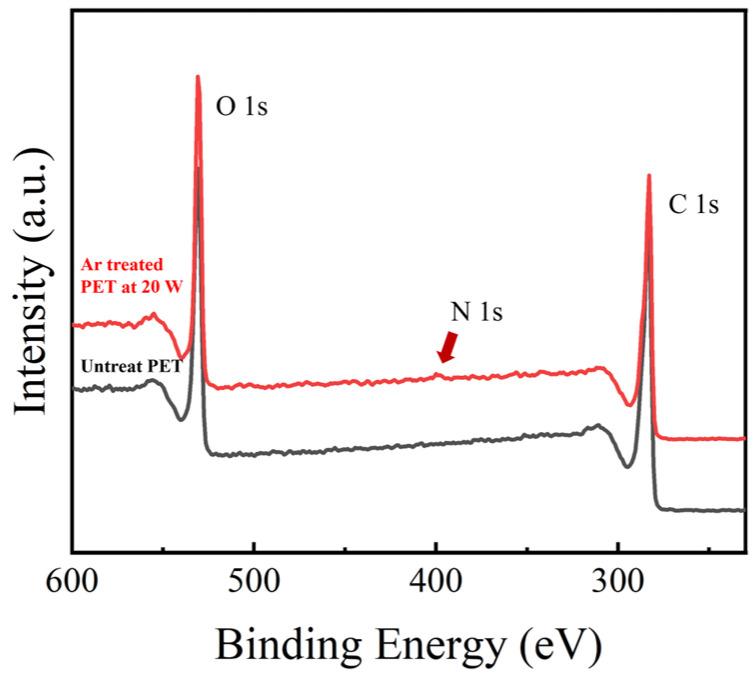
XPS spectra of PET films before and after Ar plasma treatment.

Figure 5 displays the XPS spectra of the PET films before and after Ar plasma treatment. C 1s and O 1s peaks were clearly observed on the surface of the untreated film, with atomic percentages of 73.5% and 26.5%, respectively, which was consistent with the reference reported by [25] (note that hydrogen is hardly detectable by XPS technology). After Ar plasma treatment, a weak N 1s peak was detected in addition to the C 1s and O 1s signals, originating from the reaction of plasma-generated radicals with nitrogen in the air [30]. The variations in surface elemental composition of PET under various RF power conditions are summarized in Table 4. Compared with the untreated sample, the oxygen contents were found to decrease progressively with increasing RF power, leading to a corresponding reduction in the O/C ratio. The decrease in oxygen content was a common phenomenon when PET was exposed to non-oxygen plasma environments. This effect was explained by the scission of the C-O bond, the weakest linkage in the PET polymer chain (bond energy: 324 kJ·mol^−1^), which was therefore particularly susceptible to cleavage under plasma irradiation [31]. Similar observations were documented in prior studies by [31,32].

**Figure 6:**
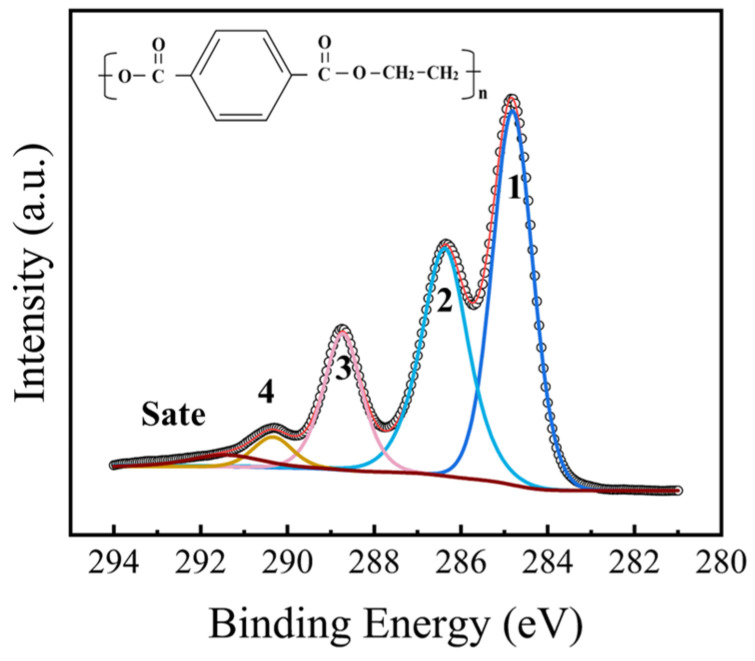
High-resolution C 1s XPS spectrum of the untreated PET sample.

The high-resolution C 1s XPS spectrum of the untreated PET sample and its corresponding peak deconvolution results are presented in Figure 6, with the detailed fitting parameters summarized in Table 5. All binding energies were calibrated with reference to the standard C 1s peak at 284.8 eV. The C 1s spectrum was successfully resolved into four main component peaks and one satellite peak, achieving a high-quality fit with an envelope residual (χ^2^) of 9. For the untreated PET, the full width at half maximum (FWHM) of the fitted peaks was relatively uniform, remaining narrowly constrained between 1.1 and 1.2 eV. The peaks located at binding energies of 284.8 eV, 286.4 eV, 288.75 eV, and 290.3 eV were assigned to the C-C/C-H, C-O, O-C=O, and O-(C=O)-O bonds, respectively [33,34]. The satellite peak observed at 291.4 eV was attributed to the π-π^⁎^ shake-up process [35]. In addition, the presence of the O-(C=O)-O component was likely attributed to carbonate-based additives, which were commonly employed to enhance melt fluidity during the commercial processing of polyester films [36].

**Figure 7:**
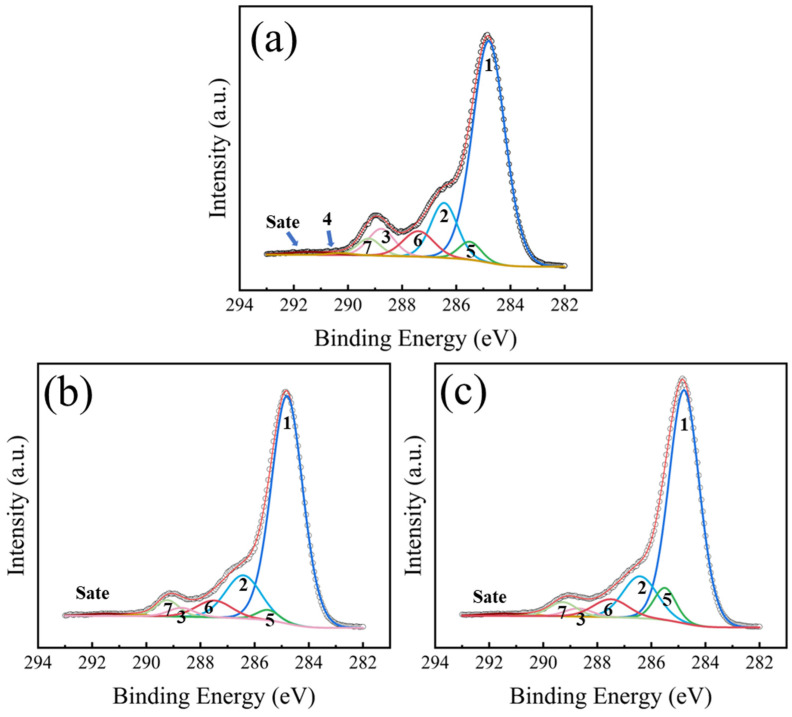
High-resolution C 1s XPS spectra of PET films treated with Ar plasma as a function of RF power: (**a**) 20 W; (**b**) 60 W; (**c**) 100 W.

The C 1s XPS spectra of the PET films treated at different RF power conditions are shown in Figure 7. The chemical state compositions and corresponding fitting parameters of the C 1s spectra after pretreatment are detailed in Table 6. Notably, in contrast to the relatively uniform peak widths of the untreated sample, the full width at half maximum (FWHM) of certain fitted peaks increased correspondingly after the plasma treatment, which was driven by the differential charging effect [33]. Furthermore, across all subsequent pretreatment conditions, the FWHM of the C 1s fitted peaks for the PET samples consistently fluctuated within a narrow margin of 0.2 eV. Compared with the untreated sample (Figure 6), three additional peaks were observed in Figure 7. The peak located at the binding energy of 285.5 eV was attributed to the C^⁎^-COO or C-N bond component (labeled C 1s (5)) [33,37]. The C 1s (6) component, located at 287.5 eV resulted from the formation of free carbonyl groups (C=O), while the peak at 289.2 eV (labeled C 1s (7)) was assigned to carboxylic acid groups (COOH) [33]. Based on the data in Table 6, as the RF power was increased from 0 W to 20 W, the relative contents of C-O, O-C=O, O-(C=O)-O, and the π-π^⁎^ shake-up satellite peak all decreased. A further increase in RF power to 100 W led to a continued decline in these components, with some peaks becoming nearly undetectable. In addition, polar functional groups such as carboxyl (COOH) and carbonyl (C=O) were introduced on the PET surface after Ar plasma treatment. However, their concentrations showed no significant change with further increases in RF power.

### 3.2. Surface Properties of PET Films After O_2_ Plasma Treatment

#### 3.2.1. Effect of O_2_ Plasma Treatment on Surface Energy of PET Films

**Figure 8:**
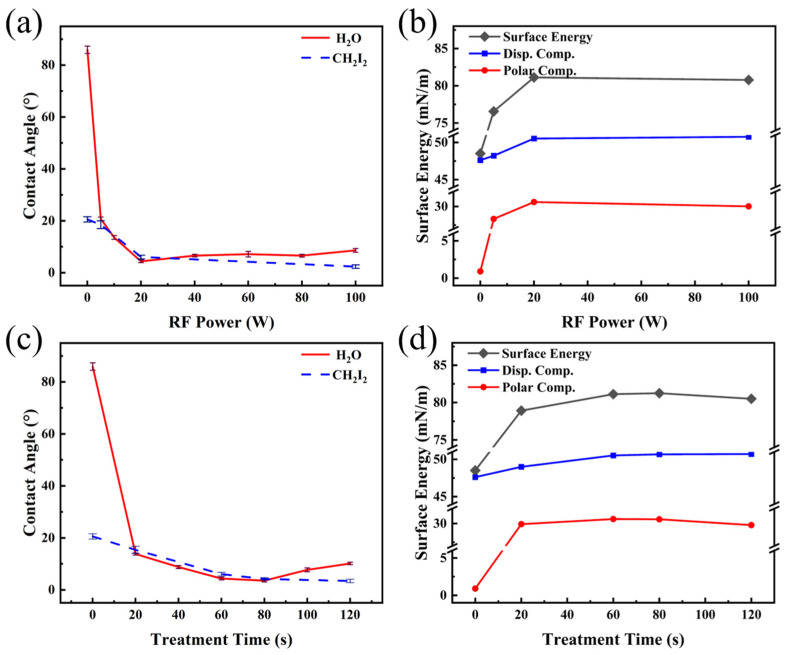
Contact angle and surface energy of PET films as a function of O_2_ plasma treatment parameters: (**a**,**b**) RF power and (**c**,**d**) treatment time.

Figure 8a presents the variation in surface wettability of PET samples treated with O_2_ plasma under different RF power levels. As illustrated, the WCA reached its minimum value of 4.4° when the RF power was increased to 20 W, indicating that a super-hydrophilic state was achieved (commonly defined as WCA below 10° [38]). A slight rebound in WCA was observed with further increase in RF power. Furthermore, DCAs were measured at three specific RF powers: 5 W, 20 W, and 100 W. The results showed that the DCA decreased continuously with increasing RF power. The corresponding surface energy, along with its dispersion and polar components derived from these data, is presented in Figure 8b. Consistent with the results observed for Ar treatment (Figure 2), the evolution of the WCA and the DCA corresponded to the variation trends of the polar component and the dispersion component of surface energy, respectively. Driven by the combined contribution of both components, the maximum surface energy of the PET film (81.12 mN·m^−1^) was achieved at the treatment power of 20 W.

At the optimal RF power of 20 W, the effect of O_2_ plasma treatment time on the wettability of PET films was systematically investigated. As shown in Figure 8c, when the treatment time was 20 s, the WCA on the PET surface decreased to 13.8°. Extending the treatment time to 80 s further reduced the WCA to 3.7°. Beyond this duration, a slight increase in the WCA was observed. In contrast, the DCA exhibited a continuous decline with prolonged treatment time. The surface energy of the PET films, including its dispersive and polar components under different treatment durations, was calculated and is included in Figure 8d. The maximum surface energy was attained at a treatment time of 80 s. Similar to the relationship between Figure 8a,b, the variations in the WCAs and DCAs were predominantly governed by the polar and dispersive components, respectively, further confirming their decisive roles in the evolution of surface wettability. The corresponding water droplet photographs are provided in Supplementary Figures S5–S8.

#### 3.2.2. Effect of O_2_ Plasma Treatment on the Topography of PET Films

**Figure 9:**
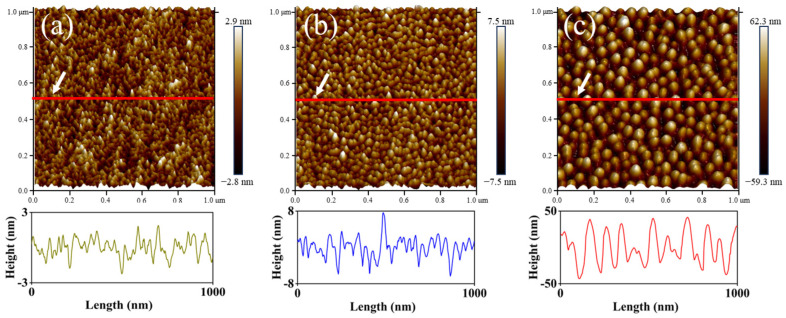
Topography of PET films treated with O_2_ plasma at different RF power levels: (**a**) 5 W; (**b**) 20 W; (**c**) 100 W.

Figure 9 displays the topography of PET samples treated with O_2_ plasma at various RF power levels. As the RF power increased, the surface microstructure became progressively more ordered, characterized by a reduction in the number of nanoscale peaks and an increase in their size. Distinct conical features were observed at 100 W and similar morphological features were also reported by Wang et al. [39]. Cross-sectional profile analysis further revealed that the height of these protrusions increased significantly with RF power, leading to a corresponding increase in surface roughness. Specifically, when the RF power was raised from 5 W to 20 W, the arithmetic average roughness (Ra) increased from 0.64 nm to 1.74 nm. A further increase in power to 100 W resulted in a roughness of 15.80 nm.

#### 3.2.3. Effect of O_2_ Plasma Treatment on the Surface Chemical State of PET Films

Table 7 summarizes the surface elemental composition of PET after O_2_ plasma treatment. Nitrogen was also detected on the treated surface, a finding consistent with the data in Table 4. Compared to the untreated sample, the treatment resulted in increased surface concentrations of oxygen and nitrogen and a corresponding decrease in carbon content, leading to a higher O/C ratio. The maximum oxygen and nitrogen contents, along with the highest O/C ratio, were achieved at the RF power of 20 W. Under this condition, the WCA reached its minimum value. Previous studies indicated that the hydrophilicity of surfaces treated with O_2_ plasma was predominantly governed by their O/C ratio [40].

**Figure 10:**
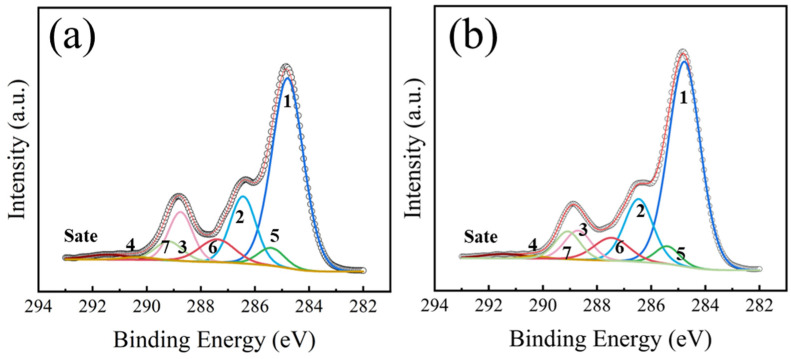
High-resolution C 1s XPS spectra of PET films treated with O_2_ plasma as a function of RF power: (**a**) 20 W; (**b**) 100 W.

Figure 10 presents the high-resolution C 1s XPS spectra of the PET surface treated with O_2_ plasma at different RF power conditions, and the corresponding quantitative analysis of the chemical bonds is summarized in Table 8. The data revealed that the chemical bond components identified in the C 1s spectrum after O_2_ plasma treatment were consistent with those observed after Ar plasma treatment. However, notable differences were found in their relative concentrations. For the same RF power, the O_2_-treated sample exhibited the higher relative concentrations of both the C=O bond and the COOH group compared to the Ar-treated sample. In addition, as the RF power was increased from 20 W to 100 W, a slight decline in the content of polar groups (C=O and COOH) was observed. This phenomenon could be attributed to the excessive etching of the O_2_ plasma on the PET surface at higher power levels [34].

### 3.3. Surface Properties of PET Films After N_2_ Plasma Treatment

#### 3.3.1. Effect of N_2_ Plasma Treatment on Surface Energy of PET Films

**Figure 11:**
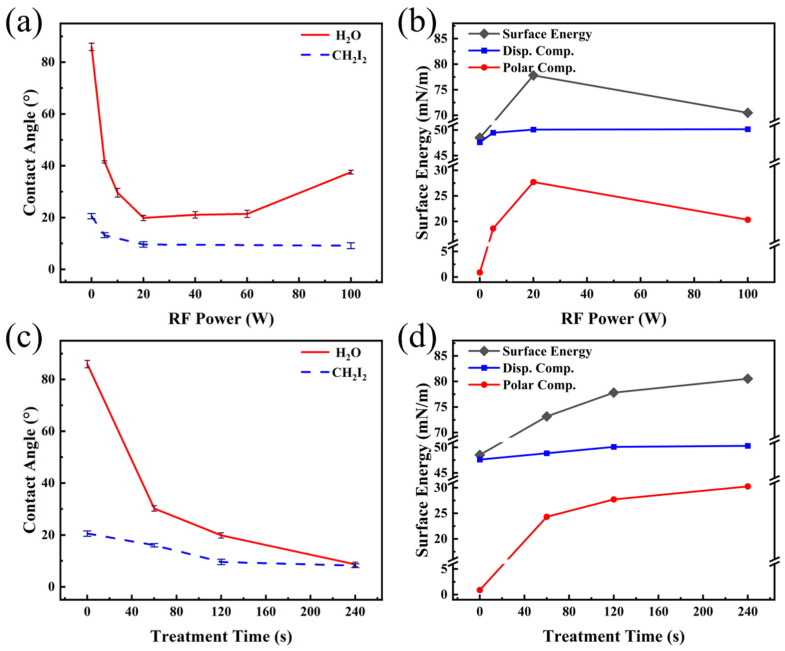
Contact angle and surface energy of PET films as a function of N_2_ plasma treatment parameters: (**a**,**b**) RF power and (**c**,**d**) treatment time.

Figure 11a shows the effect of RF power on the wettability of PET films treated with N_2_ plasma. The WCA decreased rapidly as the power increased from 0 W to 20 W, reaching a minimum of 20.6°. Further increasing the power to 60 W resulted in a slight rebound of the contact angle, while a sharp increase to 37.5° was observed at 100 W. In comparison with the results in Figure 8, the improvement in hydrophilicity achieved with N_2_ plasma treatment was significantly less pronounced than that obtained with O_2_ plasma. The DCAs were measured at three power levels: 5 W, 20 W, and 100 W. The results showed that the DCA reached its minimum at 20 W, while no significant change was observed with further increase in RF power. Figure 11b presents the variations in the surface energy of the PET film along with its dispersive and polar components. It can be seen that the polar component initially increased and then decreased with increasing RF power, reaching its maximum at 20 W. This trend was consistent with the behavior of the WCA. The dispersive component increased slightly with increasing power but stabilized beyond 20 W, which aligned with the response of the DCA. Under the combined influence of both the polar and dispersive components, the maximum surface energy of the PET film (77.81 mN·m^−1^) was achieved at a treatment power of 20 W.

The effects of N_2_ plasma treatment time on the contact angles of PET films were systematically investigated at a fixed RF power of 20 W. As shown in Figure 11c, the results indicated that the WCA continuously decreased with treatment time, reaching a minimum of 8.7° at 240 s. Similarly, the DCA decreased to its lowest value (5.8°) at the same duration. Correspondingly, the surface energy of the PET film reached a maximum of 80.53 mN·m^−1^ at 240 s. Analysis of the surface energy components (Figure 11d) further revealed that the variations in the polar and dispersive components aligned with the trends observed for the WCAs and DCAs, respectively. All optical images of the aforementioned water contact angles can be found in Supplementary Figures S9–S12.

#### 3.3.2. Effect of N_2_ Plasma Treatment on the Topography of PET Films

**Figure 12:**
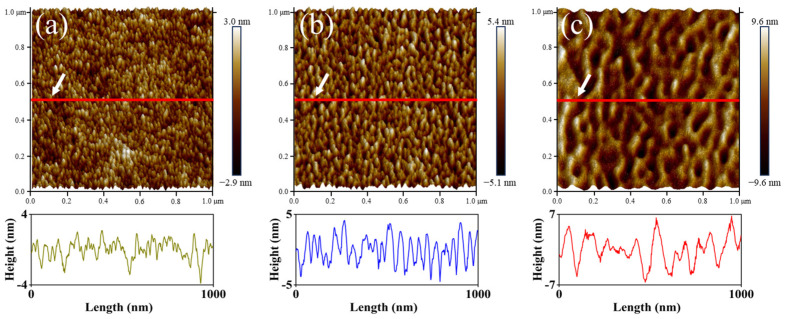
Topography of PET films treated with N_2_ plasma at different power levels: (**a**) 5 W; (**b**) 20 W; (**c**) 100 W.

Figure 12 presents the AFM topography of PET films treated with N_2_ plasma under varied RF power conditions. At the RF power of 5 W, the sample surface exhibited a fine columnar morphology. As the power was increased to 20 W, the surface features transformed into distinct ridge-like structures. Upon further elevation of the power to 100 W, a reduction in the number of ridges and an increase in their height were noted, accompanied by the gradual transition from ridge-like to dune-like morphology. Similar morphologies were also observed in the study by Junkar et al. [41,42]. The surface roughness calculated based on the AFM topographic images was 0.69 nm, 1.32 nm, and 2.30 nm for the PET films treated at 5 W, 20 W, and 100 W, respectively, indicating that the surface roughness increased continuously with increasing RF power.

#### 3.3.3. Effect of N_2_ Plasma Treatment on the Surface Chemical State of PET Films

Table 9 summarizes the evolution of the surface elemental composition of PET films treated with N_2_ plasma as a function of RF power. The data showed that as the RF power was increased, the nitrogen content on the film surface rose, while the oxygen content decreased, leading to a reduction in the O/C ratio. Similar to the case of Ar plasma treatment, the scission of the C-O bonds induced by the plasma radiation was responsible for the decrease in oxygen content.

**Figure 13:**
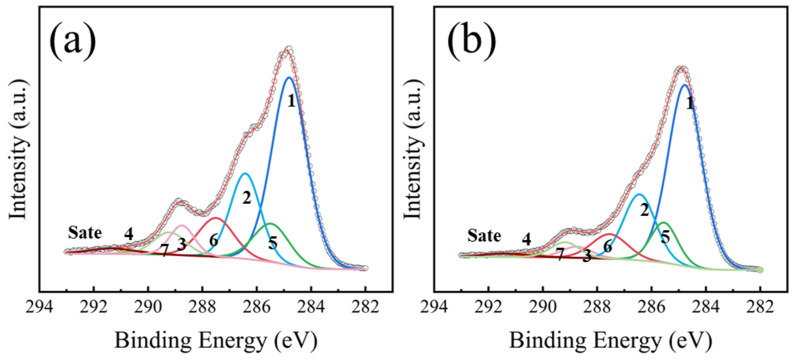
High-resolution C 1s XPS spectra of PET films treated with N_2_ plasma as a function of RF power: (**a**) 20 W; (**b**) 100 W.

Figure 13 presents the high-resolution C 1s XPS spectra and corresponding peak-fitting results of PET films treated with N_2_ plasma at 20 W and 100 W. The quantitative analysis of chemical bonds is summarized in Table 10. The chemical states formed on the PET surface after N_2_ plasma treatment were similar to those observed on O_2_-treated samples. Notably, the C 1s (6) component shifted toward higher binding energy by approximately 0.1 eV, which was attributed to the grafting of N-C=O groups onto the surface [20,41]. Due to the similar binding energies of N-C=O and C=O, these contributions could not be spectrally resolved and were therefore treated as a single component in this study. After N_2_ plasma treatment, the contents of the C-O, O-C=O, O-(C=O)-O, and the π-π^⁎^ shake-up satellite peaks were all reduced, whereas polar groups such as C=O, N-C=O, and COOH were introduced on the surface [33,41]. Furthermore, as the RF power was increased from 20 W to 100 W, the content of polar groups exhibited a decreasing trend, which was consistent with the data in Table 7 and was also attributed to excessive etching of the PET surface by the plasma under higher-power conditions [39].

## 4. Discussion

### 4.1. Relationship of WCA with the Chemical Composition of PET

As illustrated in Figure 2, Figure 8 and Figure 11, the WCAs on PET film surfaces were significantly reduced after plasma treatment in three different atmospheres compared to the untreated film. Under all conditions, the trend in WCA consistently corresponded with the evolution of the surface polar component, indicating that the increase in the polar component was the primary cause for the decrease in WCA. However, the effectiveness of three plasma treatments and the treatments’ dependence on process parameters were found to be different. In the case of Ar plasma treatment, owing to the inert nature of Ar, the principal mechanism was identified as physical etching of the polymer surface by excited Ar+ species [43]. Consequently, compared to O_2_ and N_2_ plasma treatments, less chemical modification was induced on the surface, a result supported by data in Table 5, Table 6, Table 8 and Table 10, which show that the content of polar functional groups C 1s (6) + C 1s (7) was the lowest after Ar plasma treatment. Thus, the improvement in surface hydrophilicity achieved by Ar plasma treatment remained relatively limited, and the corresponding WCAs were the highest among the three treatments (Figure 2, Figure 8 and Figure 11). Furthermore, when the RF power was increased from 20 W to 100 W, only minor changes were observed in both the WCA and the content of C 1s (6) + C 1s (7) functional groups (refer to Figure 2 and Table 6). This suggests that further elevation of power contributed little to the grafting of polar functional groups. Collectively, these results confirm that changes in surface WCA were closely related to the polar component, whose enhancement originated from an increase in surface polar functional group content.

A comparison of the results from Figure 2, Figure 8 and Figure 11 indicated that, among the three plasma atmospheres, O_2_ plasma produced the most pronounced improvement in PET surface hydrophilicity. As the RF power was increased, the WCA on the PET surface initially decreased continuously, followed by a slight rebound, while the corresponding O/C ratio likewise showed an initial rise and a subsequent decline (Table 7). Consistent with earlier reports, a higher O/C ratio was generally associated with a lower WCA [44]. Moreover, with increasing RF power, the surface polar group content also exhibited an initial increase followed by a decrease, further supporting the close correlation between the polar component and the content of polar functional groups (Table 8). When oxygen was subjected to plasma discharge, its molecules were excited, dissociated, and ionized. The reactive oxygen species then interacted with the polymer surface, promoting functionalization through the incorporation of oxygen-containing groups [20]. At lower RF powers, radicals generated on the PET surface were reacted with oxygen-derived active species (e.g., O, O_3_) to form oxygen-containing polar functional groups, which led to a reduction in the WCA [45]. As the power was increased, additional oxygen-containing groups were grafted onto the surface, further enhancing hydrophilicity. However, when the power was further raised to 100 W, decreases were observed in both the O/C ratio and the content of C 1s (6) + C 1s (7) functional groups.

The surface hydrophilicity of PET films treated with N_2_ plasma was intermediate between those treated with Ar and O_2_. When the RF power was increased, the WCA on the PET surface was observed to first decrease and then rise, while the content of C 1s (6) + C 1s (7) functional groups similarly showed an initial increase followed by a decrease. This non-monotonic variation was consistent with the behavior observed during O_2_ plasma treatment. Further support for this trend was provided by the study of Wang et al., in which PSF-PEG films were treated with Ar, O_2_, and CO_2_ plasmas over a power range of 0–100 W [46]. The minimum WCA was recorded at 60 W, followed by an increase at higher powers. The authors attributed the enhanced hydrophilicity at lower powers primarily to the incorporation of polar groups, while the decline at elevated powers was linked to pronounced surface etching. Notably, data from Table 8 and Table 10 indicated that although PET surfaces treated with N_2_ and O_2_ plasmas at identical powers had comparable contents of C 1s (6) + C 1s (7) functional groups, the WCA was significantly higher for the N_2_-treated samples. This discrepancy was primarily explained by the fact that, under N_2_ plasma conditions, the PET surface was readily functionalized by nitrogen-containing active species, forming N-C=O groups (Figure 13 and Table 10). Since the polarity of N-C=O is lower than that of C=O functional groups, the resulting surface exhibited lower hydrophilicity, which was reflected in the higher WCA.

### 4.2. Relationship of DCA with Topography of PET

The DCA was used to probe variations in the dispersion component of the surface energy, which is governed by non-polar van der Waals interactions. In untreated PET films, the surface energy was dominated by this dispersion component. After plasma treatment, however, a significant increase in surface energy was observed, primarily driven by the enhancement of the polar component. In addition, changes in topography induced by plasma treatment also contributed to the modification of the dispersion component [44,47]. Nanoscale topography acts in concert with chemical modifications to drastically shift the interfacial spreading dynamics, a phenomenon well-documented in polymer surface engineering [26]. To further explore this mechanism, the surface area difference (SAD) was calculated from three-dimensional topographic data acquired by AFM. SAD quantifies the difference between the three-dimensional surface area and its two-dimensional projected area. The calculated SAD values are summarized in Table 11. As indicated in the table, SAD increased progressively with both treatment power and duration for samples exposed to O_2_ and N_2_ plasmas. This trend was explained by the high reactivity of these plasmas, which likely promoted cross-linking on the PET surface and led to the formation of characteristic topographic features, such as conical or dune-like structures, thereby significantly increasing the three-dimensional surface area [44]. In contrast, Ar plasma exhibited lower chemical activity and appeared to induce limited cross-linking. Under identical etching conditions, Ar plasma exhibited higher energy, thereby rendering physical etching the dominant mechanism. Sustained bombardment by energetic Ar^+^ ions fragmented polymer chains on the PET surface into low-molecular-weight oxidized materials (LMWOM) [48,49]. As oxidized loose polymer fragments, LMWOM was more readily desorbed and removed by the vacuum system, leading to progressive surface flattening. Consequently, although both surface roughness and SAD were relatively elevated at low RF powers during Ar plasma treatment, they decreased as the power was further raised.

**Figure 14:**
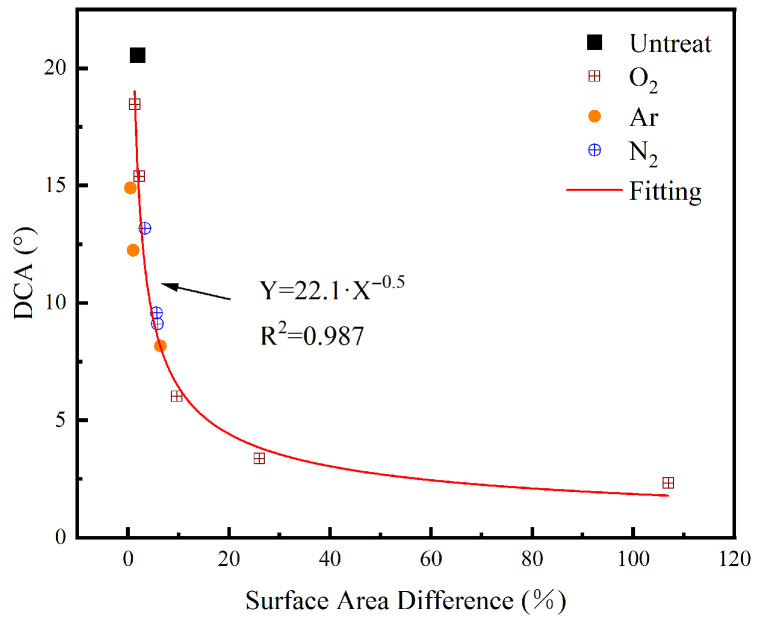
Relationship between the SAD and the DCA under various plasma treatments.

Figure 14 depicts the correlation between the DCA and the SAD for samples subjected to different plasma treatments. As shown, the DCA exhibited a negative power-law dependence on SAD, which could be described by the following expression:

(3) $$\theta \;=A\cdot {\left(\mathrm{SAD}\right)}^{n}$$

where θ denotes the DCA (°), while *A* and *n* are fitting constants. The obtained values are *A* = 22.1 and *n* = −0.5, with the coefficient of determination (R^2^) greater than 0.98.

### 4.3. Effects of Different Plasma Atmospheres and the Role of Reactive Species

To fully elucidate the distinct macroscopic wettability and topographical evolution discussed above, the fundamental roles of specific reactive species—particularly reactive oxygen species (ROS) and reactive nitrogen species (RNS)—must be considered.

For Ar plasma, the modification is primarily governed by physical bombardment rather than chemical functionalization. Energetic Ar^+^ ions induce the preferential scission of weaker C–O bonds on the PET surface [49], generating surface radicals and low-molecular-weight fragments. The subsequent desorption of these fragments leads to progressive surface etching and flattening at higher RF powers [48,50]. In stark contrast, O_2_ plasma is dominated by strong chemical reactivity driven by an abundance of ROS (e.g., O·, O_2_^+^, and O^+^) [48]. These highly reactive oxygen species efficiently interact with plasma-induced surface radicals to graft strongly polar oxygen-containing groups (such as C=O and COOH). This rapid ROS-driven functionalization significantly amplifies the polar surface energy, yielding a superhydrophilic state [51], while concurrently promoting surface cross-linking and conical micro-roughening [39].

Meanwhile, N_2_ plasma exhibits an intermediate mechanism governed by RNS (e.g., N^+^, N_2_^+^) [52]. These nitrogen-containing active species react with surface radicals to primarily form N–C=O moieties, alongside a minor generation of oxygen-containing groups (e.g., C=O and COOH) likely originating from residual oxygen or post-plasma ambient reactions. Although this combined functionalization effectively enhances hydrophilicity and induces moderate topographical reconstruction (e.g., ridge-like and dune-like structures) [42], the dominant N–C=O groups possess an intrinsic polarity inherently weaker than that of the dense ROS-derived oxygen groups [41,42]. Consequently, N_2_-treated PET surfaces exhibit slightly higher water contact angles compared to O_2_-treated counterparts.

## 5. Conclusions

In the present study, plasma treatments utilizing three different working gases (Ar, O_2_, and N_2_) were employed to modify PET. Comprehensive analyses were conducted on the water and diiodomethane contact angles of the films under varying process parameters. Subsequent investigations systematically correlated these wettability parameters with surface chemical states and morphological features. The conclusions are as follows:

1. The effects of RF plasma treatments using Ar, O_2_, and N_2_ on the surface wettability of PET films were systematically examined by correlating wettability changes with surface chemical composition and topographical evolution. Although all plasma treatments enhanced the surface hydrophilicity of PET, the optimal processing windows and dominant modification mechanisms were strongly dependent on the plasma atmosphere. The most effective conditions for wettability improvement were determined to be 60 W for 240 s (Ar), 20 W for 80 s (O_2_), and 20 W for 240 s (N_2_).
2. The evolution of WCA was primarily governed by variations in the polar component of surface energy, which originated from the introduction of polar functional groups. O_2_ plasma treatment produced the most hydrophilic surface, owing to a significant increase in the O/C ratio and the incorporation of strongly polar oxygen-containing groups (e.g., C=O and COOH). N_2_ plasma treatment resulted in a moderate enhancement due to the formation of weaker-polar nitrogen-containing groups (N-C=O), whereas Ar plasma showed limited effectiveness because surface modification was dominated by physical processes with minimal chemical functionalization.
3. Changes in the DCA were mainly associated with the dispersive component of surface energy and closely correlated with surface roughness evolution. O_2_ and N_2_ plasmas extensively altered the surface chemical structure, leading to increased SAD and roughness, while Ar plasma induced physical etching dominated by ion bombardment, resulting in surface flattening and a decrease in SAD.
4. Overall, this work demonstrates that the wettability of PET films can be effectively tuned through synergistic control of surface chemistry and topography by selecting appropriate plasma gases and processing parameters. The findings provide mechanistic insights into plasma-polymer interactions and offer a practical basis for plasma-based surface engineering of polymer materials in advanced applications.

## Figures and Tables

**Table 1:** Detailed process parameters for Ar plasma treatment.

Series	RF Power/W	Treatment Time/s
1-1	20	120
1-2	40	
1-3	60	
1-4	70	
1-5	80	
1-6	100	
1-7	60	30
1-8		60
1-9		240

**Table 2:** Detailed process parameters for O_2_ plasma treatment.

Series	RF Power/W	Treatment Time/s
2-1	5	60
2-2	10	
2-3	20	
2-4	40	
2-5	60	
2-6	80	
2-7	100	
2-8	20	20
2-9		40
2-10		80
2-11		100
2-12		120

**Table 3:** Detailed process parameters for N_2_ plasma treatment.

Series	RF Power/W	Treatment Time/s
3-1	5	120
3-2	10	
3-3	20	
3-4	40	
3-5	60	
3-6	100	
3-7	60	60
3-8		240

**Table 4:** Surface composition of PET films before and after Ar plasma treatment (at.%).

Treatment Parameters				C	N	O	O/C	WCA/°
Gas	Power/W	Time/s	Pressure/Pa					
Un-treated				73.5	—	26.5	0.36	85.9 ± 1.5
Ar	20	120	1	76.2	1.3	22.6	0.30	47.5 ± 1.7
	60			83.3	1.2	15.4	0.18	43.0 ± 0.9
	100			85	1	14	0.16	46.4 ± 0.7

**Table 5:** Chemical bonding states of untreated PET films derived from the deconvoluted C 1s XPS spectra.

C 1s	1	2	3	4	Satellite	χ^2^
	C-C/C-H	C-O	O-C=O	O-C=O-O	π-π^⁎^	
Binding Energy (eV)	284.8	286.4	288.75	290.3	291.4	
FWHM(eV)	1.2	1.2	1.1	1.1	2	
Reference	[33,35]	[33,35]	[33,35]	[34]	[35]	
Untreated	44.15%	33.52%	15.28%	3.43%	3.62%	9

**Table 6:** Quantitative analysis of chemical bonding states in PET films subjected to Ar plasma treatment.

C 1s	1	2	3	4	5	6	7	Satellite	C 1s (6) + C 1s (7)	χ^2^
	C-C/C-H	C-O	O-C=O	O-C=O-O	C^⁎^-COO /C-N	C=O	COOH	π-π^⁎^		
Binding Energy (eV)	284.8	286.4	288.75	290.3	285.5	287.5	289.2	291.4		
FWHM(eV)	1.5	1.3	1.1	1.1	1.2	1.5	1.4	2		
Reference	[33,35]	[33,35]	[33,35]	[34]	[28,32]	[33]	[33]	[35]		
20 W	65.19%	13.04%	6.49%	0.65%	3.25%	6.94%	3.15%	1.30%	10.09%	8
60 W	70.92%	14.89%	2.13%	0.00%	2.13%	5.67%	3.55%	0.71%	9.22%	8
100 W	67.57%	13.51%	2.03%	0.00%	6.76%	6.08%	3.38%	0.68%	9.46%	11

**Table 7:** Surface composition of PET films after O_2_ plasma treatment (at.%).

Treatment Parameters				C	N	O	O/C	WCA/°
Gas	Power/W	Time/s	Pressure/Pa					
O_2_	5	60	1	72	1.1	26.9	0.37	20.7 ± 0.7
	20			69.4	1.4	29.2	0.42	4.4 ± 0.5
	100			71.0	1.0	28.0	0.39	8.6 ± 0.7

**Table 8:** Quantitative analysis of chemical bonding states in PET films subjected to O_2_ plasma treatment.

C 1s	1	2	3	4	5	6	7	Satellite	C 1s (6) + C 1s (7)	χ^2^
	C-C/C-H	C-O	O-C=O	O-C=O-O	C^⁎^-COO /C-N	C=O	COOH	π-π^⁎^		
Binding Energy (eV)	284.8	286.4	288.75	290.3	285.5	287.5	289.2	291.4		
Reference	[33,35]	[33,35]	[33,35]	[34]	[28,32]	[33]	[33]	[35]		
20 W	57.80%	15.61%	6.94%	0.58%	4.05%	6.94%	6.36%	1.73%	13.29%	4
100 W	54.64%	15.85%	10.38%	0.55%	4.37%	7.10%	4.92%	2.19%	12.02%	6

**Table 9:** Surface composition of PET films after N_2_ treatment (at.%).

Treatment Parameters				C	N	O	O/C	WCA/°
Gas	Power/W	Time/s	Pressure/Pa					
N_2_	5	120	1	70.9	9.2	19.9	0.28	41.5 ± 0.4
	20			74.1	9.4	16.5	0.22	20.6 ± 1.0
	100			77.7	9.6	13.2	0.17	37.5 ± 0.7

**Table 10:** Quantitative analysis of chemical bonding states in PET films subjected to N_2_ plasma treatment.

C 1s	1	2	3	4	5	6	7	Satellite	C 1s (6) + C 1s (7)	χ^2^
	C-C/C-H	C-O	O-C=O	O-C=O-O	C^⁎^-COO /C-N	C=O /N-C=O	COOH	π-π^⁎^		
Binding Energy (eV)	284.8	286.4	288.75	290.3	285.5	287.6	289.2	291.4		
Reference	[33,35]	[33,35]	[33,35]	[34]	[27,32]	[33,41]	[33]	[35]		
20 W	48.54%	18.45%	5.34%	0.49%	9.71%	10.19%	5.34%	1.94%	15.53%	7
100 W	55.56%	18.89%	2.78%	0.56%	9.44%	7.78%	3.89%	1.11%	11.67%	8

**Table 11:** Effect of processing parameters on the surface roughness and the SAD of plasma-treated PET films.

Sample			DCA/°	Ra/nm	SAD/%
Gas	Power/W	Time/s			
Untreated			20.90	0.58	1.93
Ar	20	120	8.2	1.99	6.41
	60		12.2	1.01	1.02
	100		14.9	0.63	0.51
O_2_	5	60	18.5	0.64	1.32
	20		6.0	1.74	9.65
	100		2.3	15.80	107.00
	20	20	15.4	0.88	2.23
	20	120	3.4	4.04	26.00
N_2_	5	120	13.2	0.69	3.32
	20		9.6	1.32	5.67
	60		9.1	2.30	5.85

## Data Availability

Data available on request from the corresponding authors.

## Supplementary Materials

The following supporting information can be downloaded at: https://www.mdpi.com/article/10.3390/nano16100615/s1, Figures S1–S4 show optical images of water and diiodomethane droplets on PET surfaces treated at different applied RF powers and treatment times in Ar plasma. Figures S5–S8 show optical images of water and diiodomethane droplets on PET surfaces treated at different applied RF powers and treatment times in O_2_ plasma. Figures S9–S12 show optical images of water and diiodomethane droplets on PET surfaces treated at different applied RF powers and treatment times in N_2_ plasma.

## Abbreviations

The following abbreviations are used in this manuscript:

WCA	Water Contact Angle
DCA	Diiodomethane Contact Angles
LMWOM	Low-Molecular-Weight Oxidized Materials
SAD	Surface Area Difference
