# Supplementary material for: Plasma–Induced Modification Mechanisms of PET Films: Correlated Evolution of Topographical Features and Surface Chemical States
Source: Nanomaterials (Basel). 2026 May 17;16(10):615. doi: 10.3390/nano16100615 (PMC13209316; doi:10.3390/nano16100615)
Supplement: Supplementary file 1 [file nanomaterials-16-00615-s001.zip › nanomaterials-4228901-supplementary.pdf]

Supplemental Information: Plasma–Induced Modification Mechanisms of PET Films: Correlated Evolution of Topographical Features and Surface Chemical States

Supporting Information

Figure S1. Optical images of water droplets on PET surfaces treated at different applied RF powers in Ar plasma.

Figure S2. Optical images of diiodomethane droplets on PET surfaces treated at different applied RF powers in Ar plasma.

Figure S3. Optical images of water droplets on PET surfaces treated at different treatment times in Ar plasma.

Figure S4. Optical images of diiodomethane droplets on PET surfaces treated at different treatment times in Ar plasma.

Figure S5. Optical images of water droplets on PET surfaces treated at different applied RF powers in O_2 plasma.

Figure S6. Optical images of diiodomethane droplets on PET surfaces treated at different applied RF powers in O_2 plasma.

Figure S7. Optical images of water droplets on PET surfaces treated at different treatment times in O_2 plasma.

Figure S8. Optical images of diiodomethane droplets on PET surfaces treated at different treatment times in O_2 plasma.

Figure S9. Optical images of water droplets on PET surfaces treated at different applied RF powers in N_2 plasma.

Figure S10. Optical images of diiodomethane droplets on PET surfaces treated at different applied RF powers in N_2 plasma.

Figure S11. Optical images of water droplets on PET surfaces treated at different treatment times in N_2 plasma.

Figure S12. Optical images of diiodomethane droplets on PET surfaces treated at different treatment times in N_2 plasma.

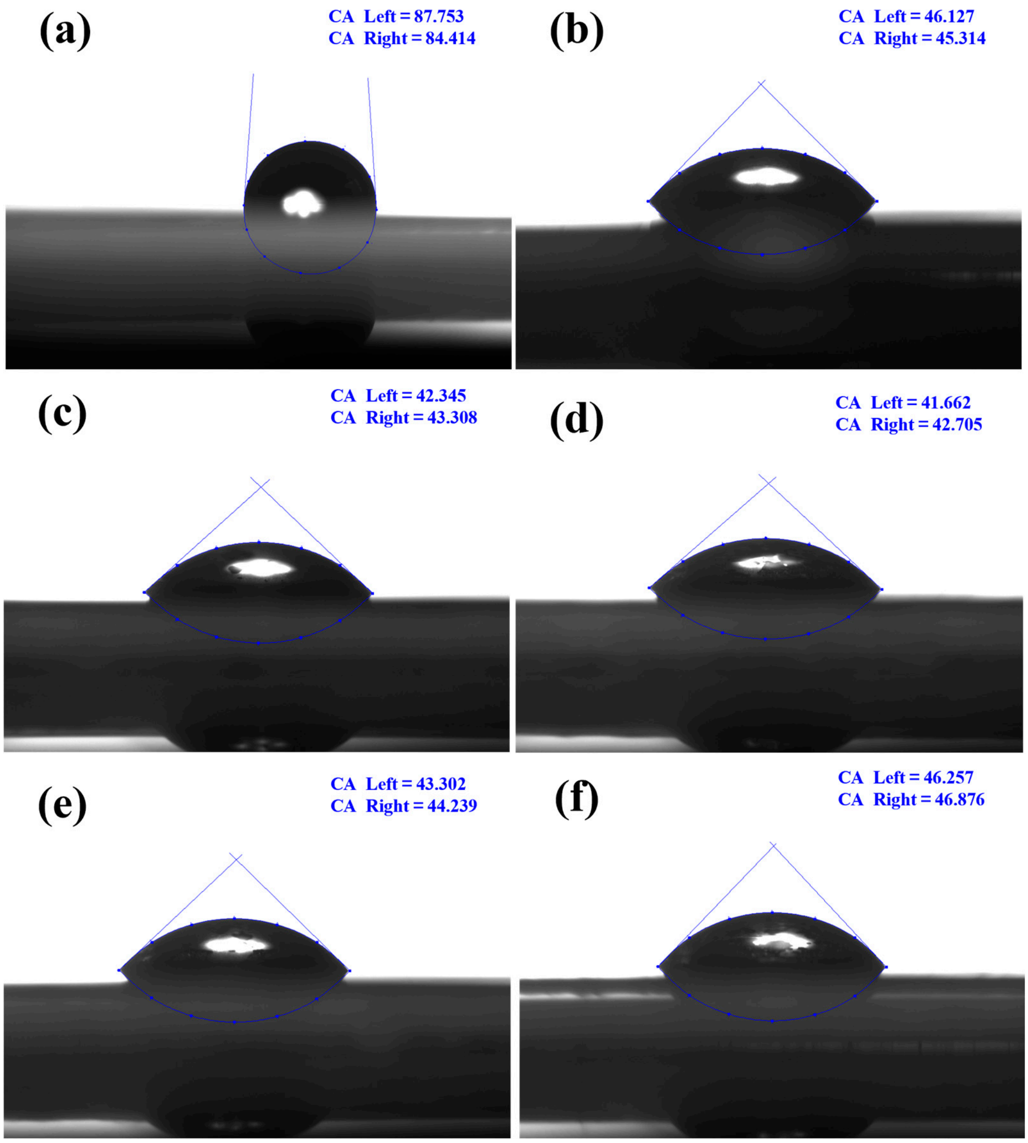

Figure S1. Optical images of water droplets on PET surfaces treated at different applied RF powers in Ar plasma: (a) untreated; (b) 20 W; (c) 40 W; (d) 60 W; (e) 80 W; (f) 100 W.

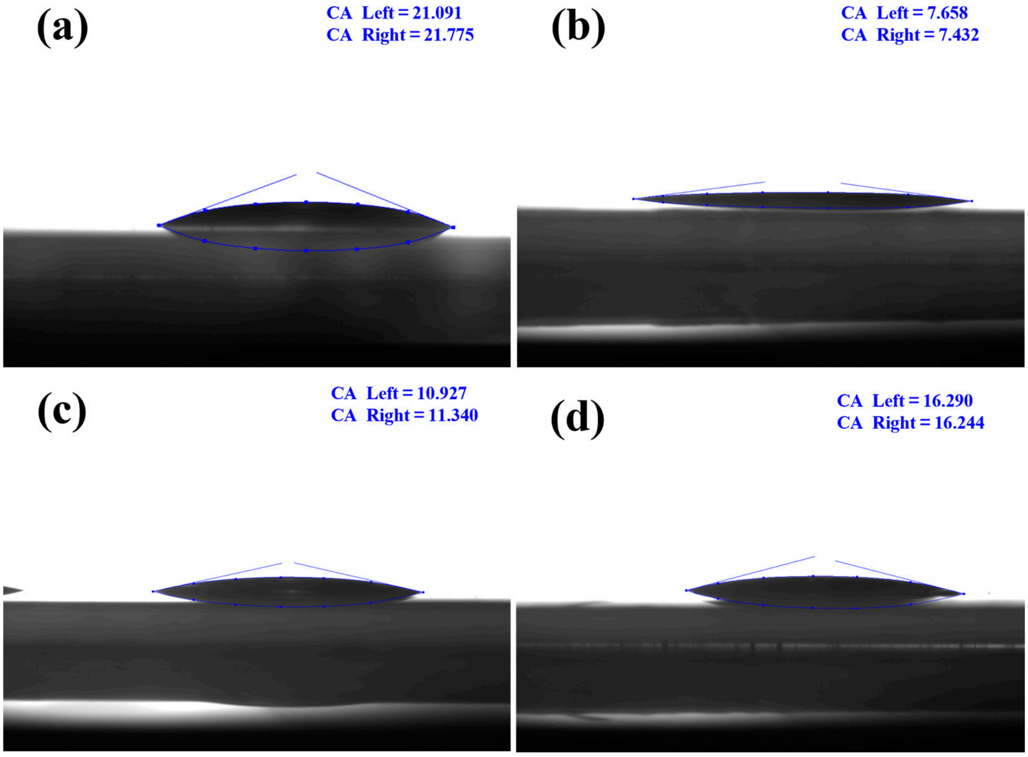

Figure S2. Optical images of diiodomethane droplets on PET surfaces treated at different applied RF powers in Ar plasma: (a) untreated; (b) 20 W; (c) 60 W; (d) 100 W.

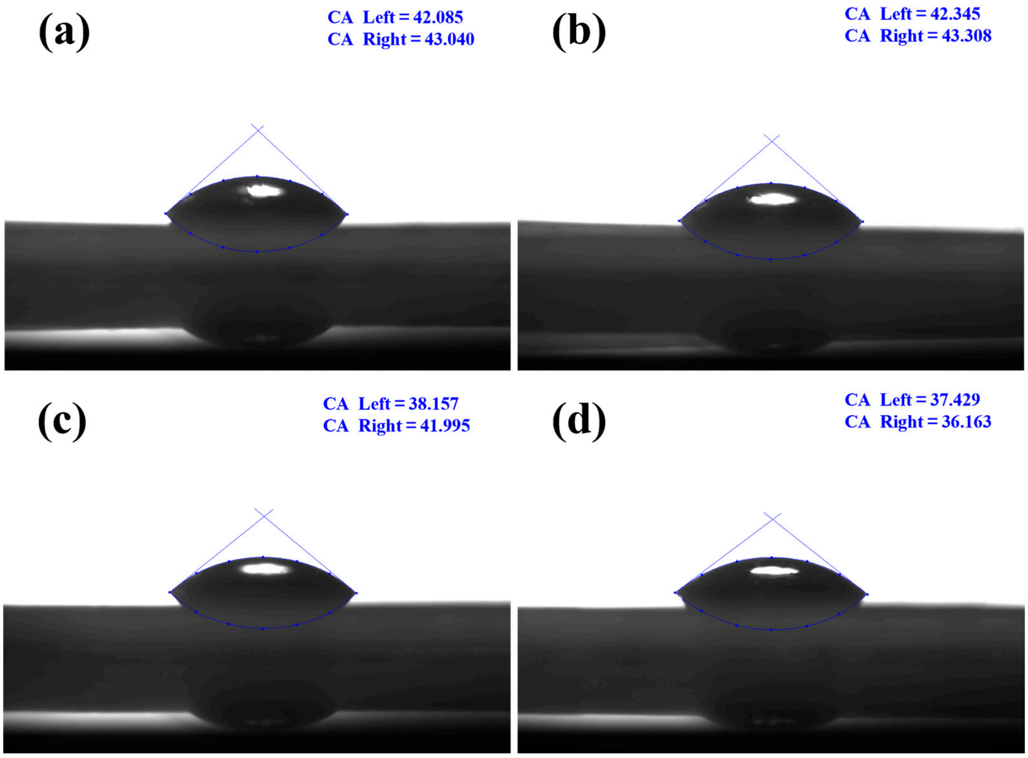

Figure S3. Optical images of water droplets on PET surfaces treated at different treatment times in Ar plasma: (a) 60 s; (b) 120 s; (c) 180 s; (d) 240 s.

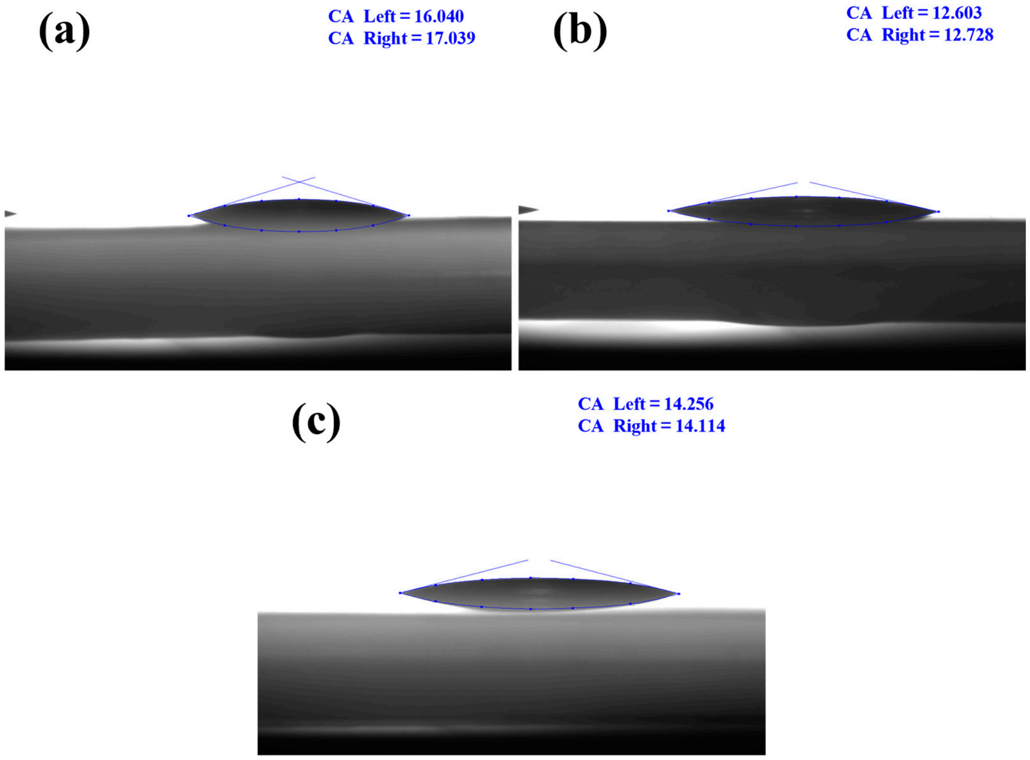

Figure S4. Optical images of diiodomethane droplets on PET surfaces treated at different treatment times in Ar plasma: (a) 60 s; (b) 120 s; (c) 240 s.

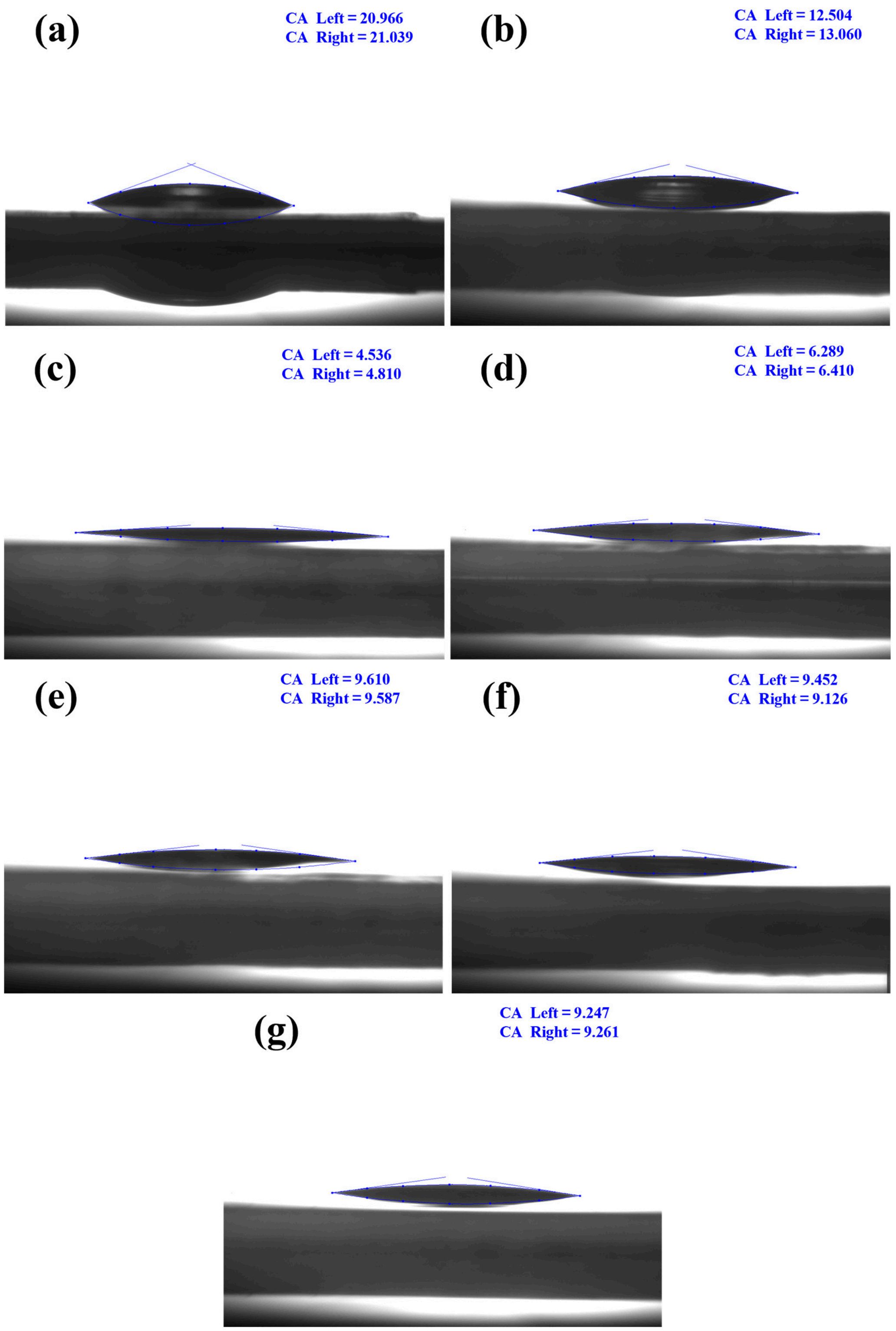

Figure S5. Optical images of water droplets on PET surfaces treated at different applied RF powers in O_2 plasma: (a) 5 W; (b) 10 W; (c) 20 W; (d) 40 W; (e) 60 W; (f) 80 W; (g) 100 W.

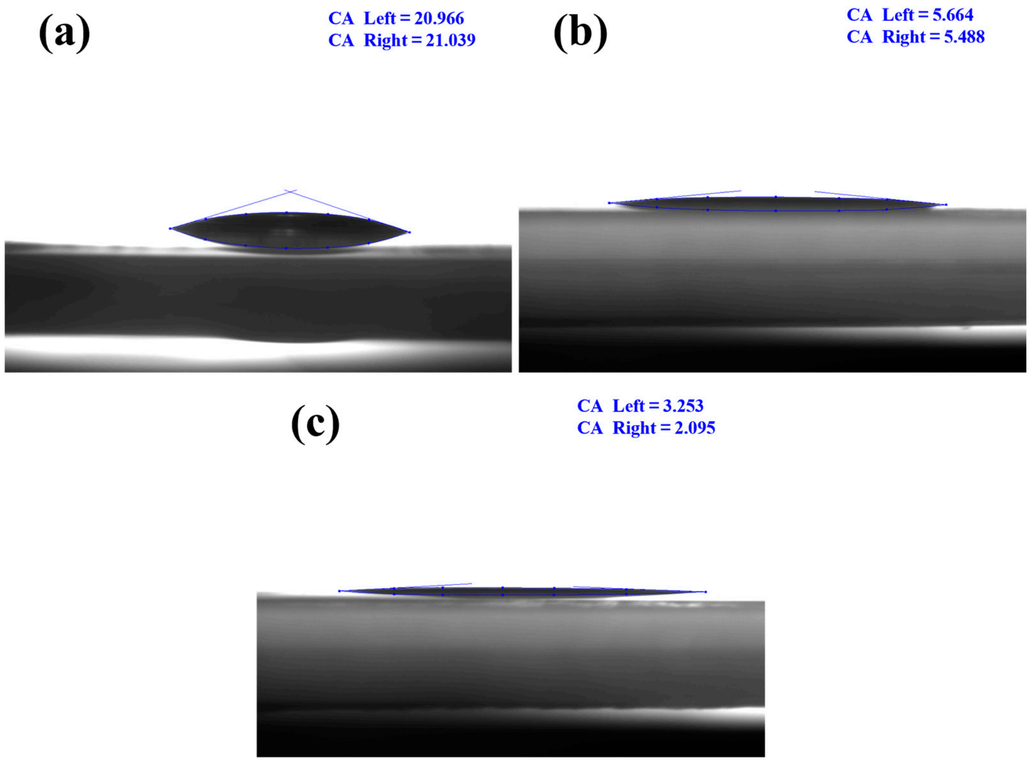

Figure S6. Optical images of diiodomethane droplets on PET surfaces treated at different applied RF powers in O_2 plasma: (a) 5 W; (b) 20 W; (c) 100 W.

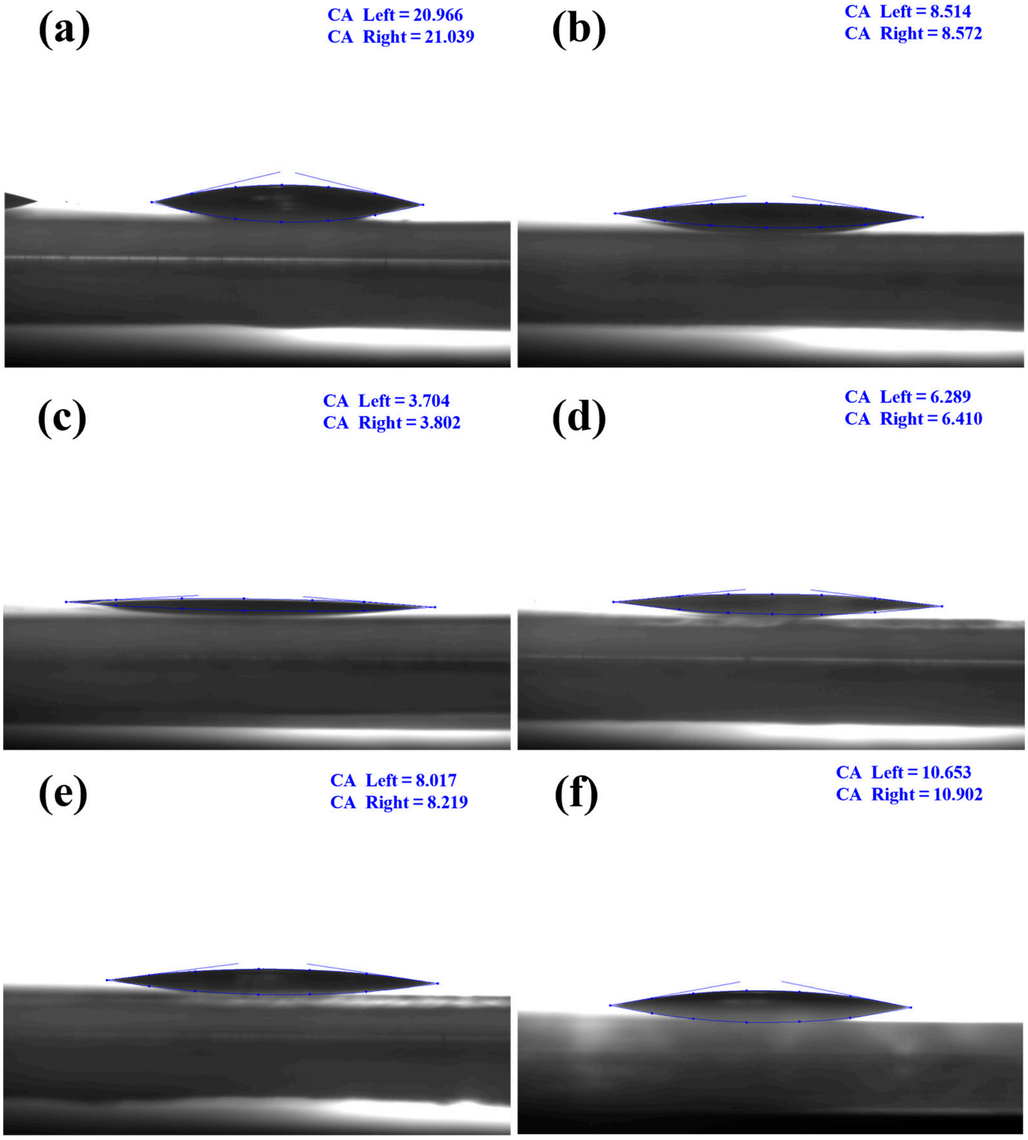

Figure S7. Optical images of water droplets on PET surfaces treated at different treatment times in O_2 plasma: (a) 20 s; (b) 40 s; (c) 60 s; (d) 80 s; (e) 100 s; (f) 120 s.

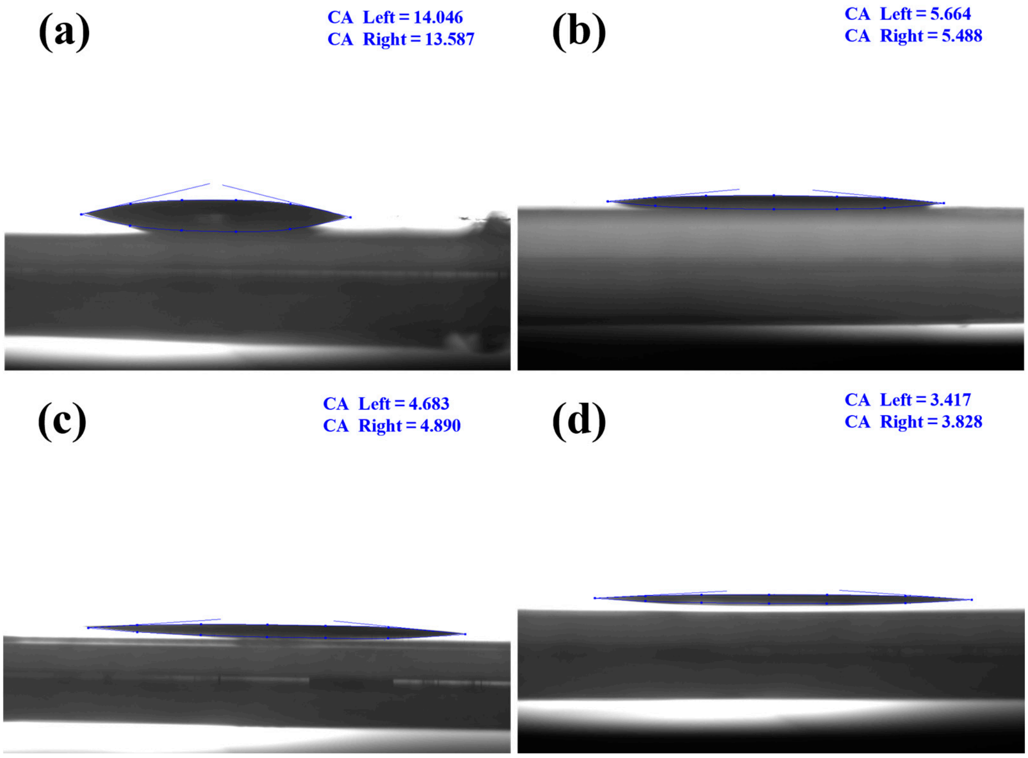

Figure S8. Optical images of diiodomethane droplets on PET surfaces treated at different treatment times in O_2 plasma: (a) 20 s; (b) 60 s; (c) 80 s; (d) 120 s.

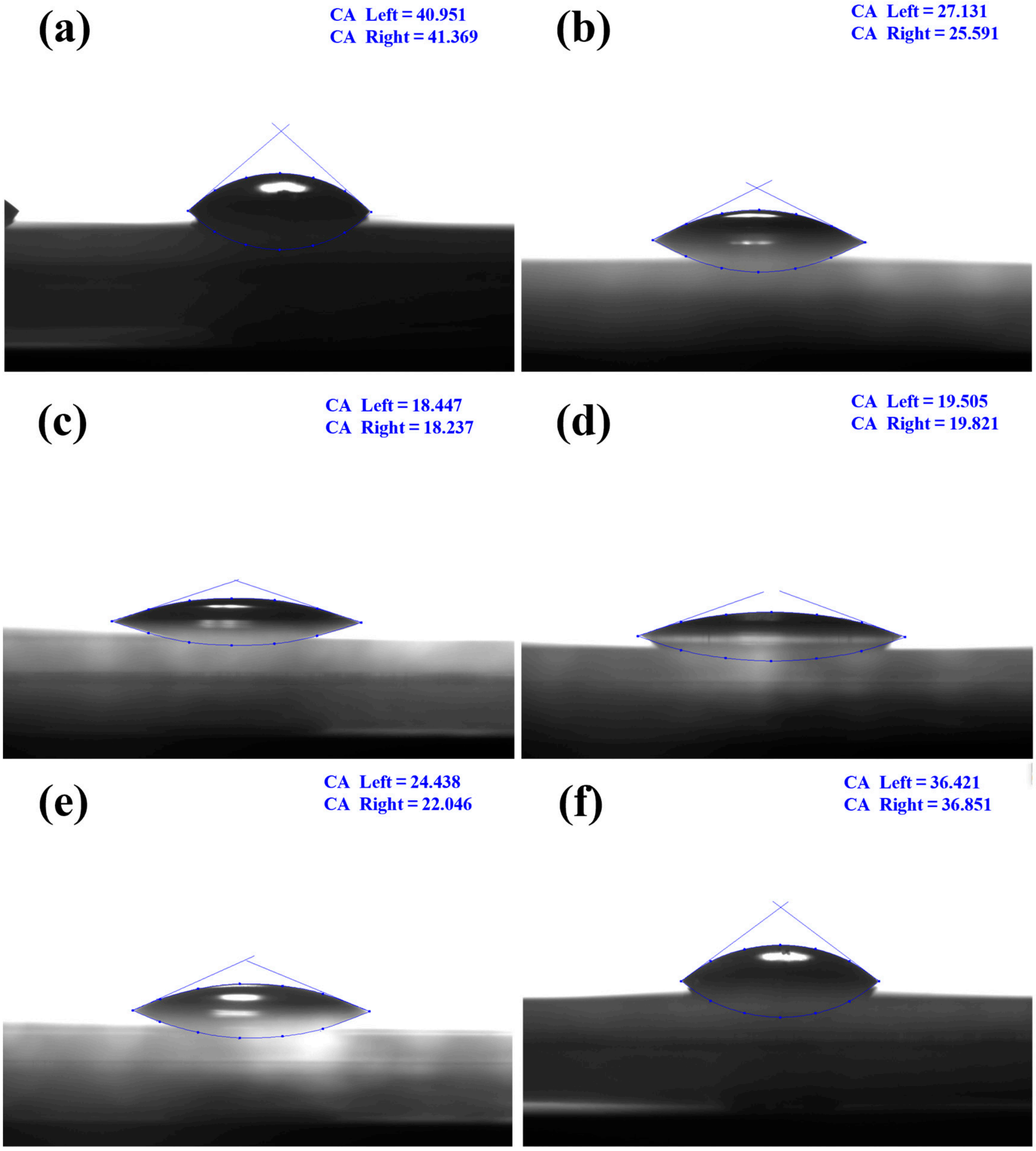

Figure S9. Optical images of water droplets on PET surfaces treated at different applied RF powers in N_2 plasma. : (a) 5 W; (b) 10 W; (c) 20 W; (d) 40 W; (e) 60 W; (f) 100 W.

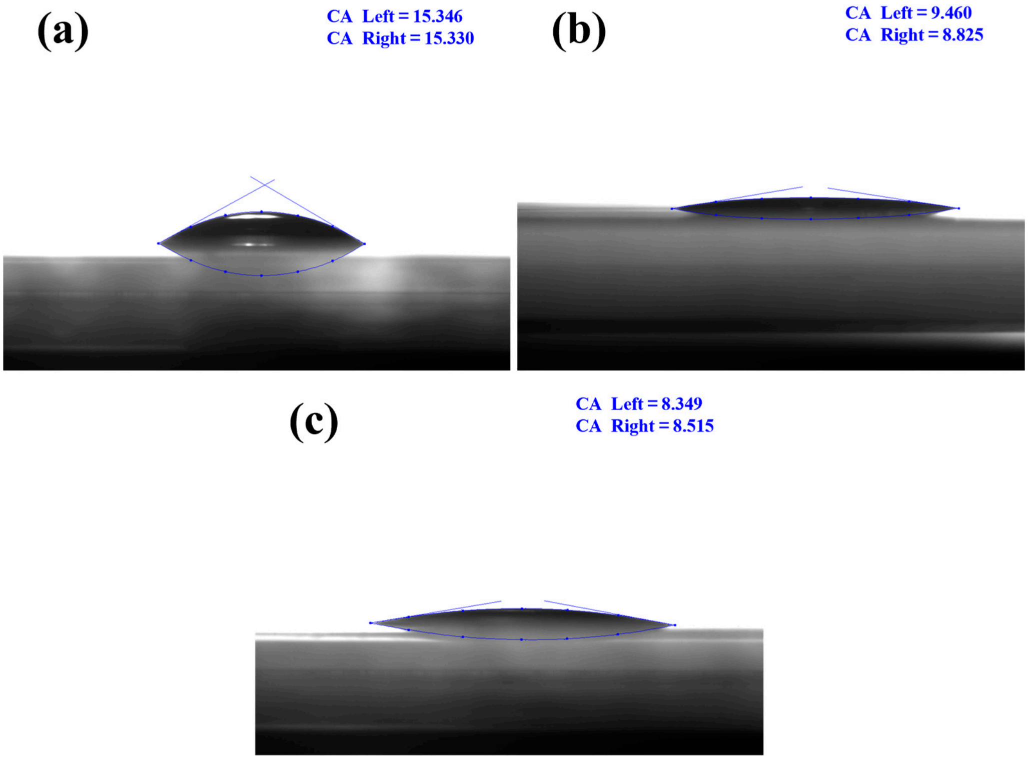

Figure S10. Optical images of diiodomethane droplets on PET surfaces treated at different applied RF powers in N_2 plasma: (a) 5 W; (b) 20 W; (c) 100 W.

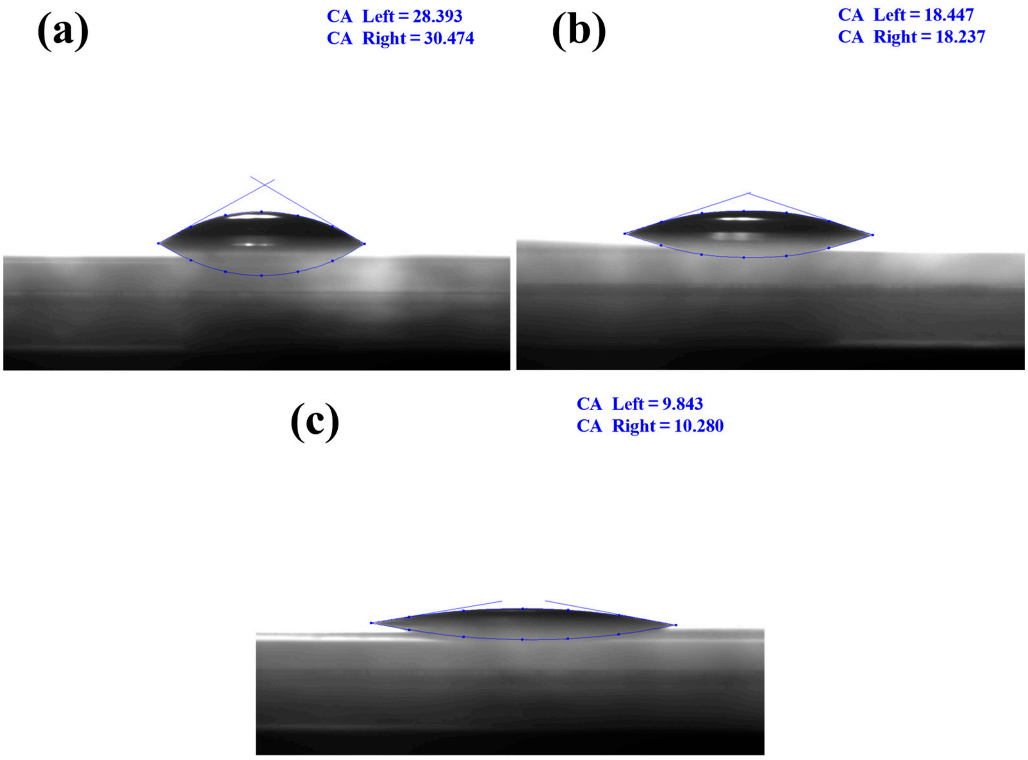

Figure S11. Optical images of water droplets on PET surfaces treated at different treatment times in N_2 plasma: (a) 60 s; (b) 120 s; (c) 240 s.

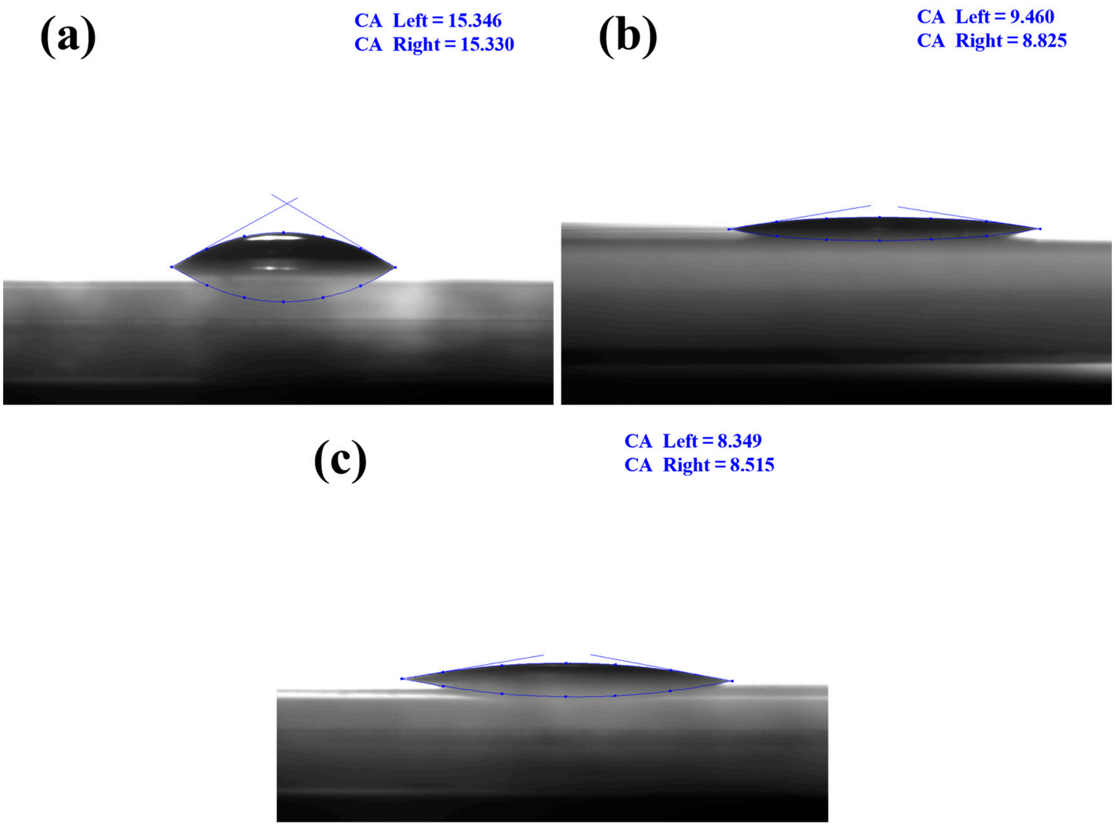

Figure S12. Optical images of diiodomethane droplets on PET surfaces treated at different treatment times in N_2 plasma: (a) 60 s; (b) 120 s; (c) 240 s.
